# Restoration of IL-11 and IL-15 cytokine production post calcium modulators and ROS treatment can assist viral clearance both* in vitro* and *in vivo*

**DOI:** 10.22038/IJBMS.2022.65983.14536

**Published:** 2023-02

**Authors:** Kehkshan Jabeen, Aneela Javed, Asim Waris, Shaheen Shahzad

**Affiliations:** 1 Genomics Research Lab, Department of Biological Sciences, International Islamic University Islamabad, Islamabad, Pakistan; 2 Healthcare Biotechnology, Atta-ur Rahman School of Applied Biosciences (ASAB), National University of Sciences and Technology (NUST), H-12 Campus, Islamabad, Pakistan; 3 School of Mechanical & Manufacturing Engineering (SMME), National University of Sciences and Technology (NUST), H-12 Campus, Islamabad, Pakistan

**Keywords:** Hepatitis B virus, Hepatocellular carcinoma, Interleukin-11, Interleukin-15, Reactive oxygen species

## Abstract

**Objective(s):**

Hepatitis B virus (HBV) infection alters the cytokines production to establish persistent infection. A reversion of cytokines back to their normal state can be a promising therapeutic approach to establish an optimal host immune response.

**Materials and Methods:**

We investigated the alteration in expression of IL-15 and IL-11 after HBV infection *in vitro* and *in vivo* in PBMCs of 63 individuals divided into various HBV-infected patient groups. The mRNA expression was evaluated post-anti-oxidant and calcium modulators treatment by Real-time qPCR.

**Results:**

*In vitro* mRNA expression of both cytokines, post-infection was down-regulated considerably. Interestingly, in line with* in vitro* results, both cytokines’* in vivo* expression was intensively down-regulated in chronic HBV-infected individuals rather than healthy controls. Both cytokines’ expression was up-regulated in cases of recovery compared with the inactive carriers and chronic HBV-infected individuals. IL-15 mRNA expression was significantly up-regulated in both cell lines post EGTA and Ru360 treatment while a significant increase was observed in the HepAD38 cell line with NAC and BAPTA treatment. IL-11 mRNA expression was significantly up-regulated in the HepG2 cell line after all modulator treatments, whereas in the HepAd38 cell line it was observed after BAPTA treatment. Our results thus indicate that viral infection tends to down-regulate the expression of cytokines and an* in vivo* up-regulation is an indication of recovery.

**Conclusion:**

Treatment of anti-oxidants and calcium modulators has resulted in the successful restoration of these cytokines thus pointing towards the use of calcium modulators to boost natural antiviral cytokine production.

## Introduction

Hepatitis B virus (HBV) is one of the potentially lethal agents of chronic liver infection and is deemed a major health burden worldwide. World Health Organization reported 296 million individuals were living with HBV globally in 2019 (1). The worldwide burden of primary liver cancer (LC) and cirrhosis have amplified from 2012 to 2017. In this regard, viral hepatitis is the utmost cause of liver deaths. Moreover, nonalcoholic fatty liver disease (NAFLD) (2) is a highly rapid growing contributor to liver mortality and morbidity.

The prospects of HBV infection are linked through peculiarities of anti-HBV immunity (3). It is a well-known fact that adaptive immune cells are implicated in the pathogenesis of hepatic inflammation, and T cells significantly contribute as antiviral defense in chronic liver damage (3). Recruitment of different innate and adaptive cells, like monocytes, neutrophils, and diverse effectors cells, to the sites of tissue damage or infection may be possible through the release of inflammatory chemokines by a diverse cell group of immune cells (4). Thus cytokines are a vital part of the diverse and critical modulators of immune function (5). Cytokine patterns, present in serum are widely associated with HBV viral load and persist by the extent of inflammation in chronic hepatitis (3). Thus serum cytokine levels and changes in these cytokines during the course of infection are critical to deciding the fate of infection and viral clearance.

IL-15 and IL-11 are key cytokines reported to take a critical part in the regulation of infection and inflammation. Accessibility of IL-15 can ramp up or dampen down both innate and adaptive immune responses, respectively (6, 7). Virally induced IL-15 expression is important for the proliferation, survival, and activation, of immune cells (NK cells, CD8+ memory T-cells, and γδ T-cells) into functional effectors, which are able to efficiently eliminate the virus. IL-11, being a member of the IL-6 family, plays an important role in decreasing the pro-inflammatory cytokines and is found to be effective in inhibiting the inflammatory responses of chronic liver. Activities of macrophages are controlled by IL-11, by blocking the transcription factor NF-κB (8-11). Thus, it is worth quantifying the expression of these cytokines during the course of HBV infection and the effect of various modulators (calcium modulators and anti-oxidants) on the expression of these specific cytokines. 

HBV expression is linked with physiological variations including calcium homeostasis disturbance along with ROS level up-regulation which prop up mitochondrial damage and dysfunction (12, 13). An ideal mechanism, intracellular Ca^+2^ signaling modulation, is used to create a permissive cellular environment for viruses due to the versatility in the nature of Ca^+2^ signaling. In HBV replicating cells, signaling of altered Ca^+2^ and elevated Ca^+2 ^are noticed too (14). Thus, we hypothesize that reversing ROS production and Ca^+2 ^deregulation must have an affirmative impact on the clearance or control of HBV by altering various cytokine expressions most likely. 

Hypothesizing that HBV-induced inflammation has important implications in the production of these cytokines. The recent study evaluates HBV infection’s effect on IL-15 and IL-11 expression both *in vitro *and* in vivo*. Furthermore, changes in the aforementioned cytokine expression in both pre and post anti-oxidant (NAC) and calcium modulators (EGTA-AM, BAPTA-AM, and Ru360) treatment were also evaluated *in vitro *to evaluate if these modulators can be used for future therapeutics against HBV infection.

## Materials and Methods

***Cell lines and plasmids***

HepG2 human hepatoma cell line was purchased from the American Type Culture Collection (ATCC), and cells were maintained as described previously (15). Dr. Jing-hsiung James Ou (University of Southern California) contributed pHBV1.3mer DNA encoding wild-type HBV genome generously. HepAD38 cell line was supplied by Dr. Christoph Seeger (Philadelphia, PA, USA) (16). According to earlier reports (15, 16), tetracycline-resistant HepAD38 cells harboring the entire HBV genome were sustained. Using Trans IT^®^-LT1 transfection reagent (Mirus; Madison, WI, USA), the plasmid (pHBV1.3mer) (300 ng) encoding 1.3merHBVgenome transfected the HepG2 cell line transiently. We have grown HepG2 and HepAD38 cells with or without NAC (Millipore, Sigma, MO, USA), BAPTA-AM (Abcam: Cambridge, MA, USA), EGTA -AM (Calbiochem CA, USA), and Ru360 (EMD Millipore Corp; Billerica, MA, USA) to block Ca^+2^ uptake into mitochondria (15). 

***Ethical statement***

International Islamic University’s Departmental Ethical Committee approved the study (Letter No. IIUI/FBAS/BIO.SCI/01). The enrolled patients with legal guardians provided assent for their participation in the study, and they were assured that the data would be kept confidential. 

***Study subjects***

 Researchers divided sixty-three participants into four groups namely controls (n=29), inactive carriers (n=5), recovered cases (n=9), and chronic hepatitis B patients (n=20). Subjects such as chronic hepatitis B patients, inactive carriers, and recovered cases were collected from the Holy Family Hospital Rawalpindi, Pakistan while for the control group blood samples were provided by healthy individuals. 

Multiple studies (17-25) have described the inclusion criteria of the selected individuals as per international criteria. The following criteria were also given in our previous study (15): For inactive HBV carriers: 1) medical history of HBsAg positive > 4 years, 2) HBeAg negative, anti-HBe antibody positive, 3) no clinically proven liver disease symptoms, 4) serum HBV below 10^5^ IU/ml DNA; for chronic hepatitis B subjects: 1) HBsAg positive >6 months or longer, 2) serum HBV DNA >20,000 IU/ml (in HBeAg positive patients), and 3) Serum HBV DNA between 2000 and 20,000 IU/ml (HBeAg-negative patients); recovered cases: 1) HBsAg-negative patients, 2) hepatitis B core antibody (antiHBc) positive, 3) anti-HBs positive. Patients were excluded in the study that did not fulfill the above-given criteria. A few controlled samples were taken from healthy blood donors. The criterion for the controlled subjects was that they had evidence of having been infected with hepatitis B, including anti-HBs, HBcAg, or anti-HBc positive results, and that their serum HBV DNA levels were undetectable (15).

***Extraction of RNA***

In *in vitro* experiments, the QiagenRNeasy® mini kit was used for RNA extraction from cell lines as the manufacturer specified. Extraction of RNA from PBMCs was done by the Trizol method for *in vivo* experiments (26).

***cDNA Synthesis***

Superscript III First-Strand Synthesis SuperMix made it possible to synthesize complementary DNAs for *in vitro* studies. Similarly, the use of Moloney Murine Leukemia Virus Reverse Transcriptase (M-MLV RT) (Invitrogen, Cat No: 28025013) made it possible to synthesize cDNA according to the manufacturer’s instructions for *in vivo* study. For more downstream experimentation, the cDNA was diluted 1:10.

***Real-time qRT-PCR***

IL-11 and IL-15RNA levels were quantified using the ΔΔCt method by using the DyNAmo HS SYBR Green qPCR kit with real-time qRT-PCR. Primer3 software was used to design primers for the target genes. Gradient PCR optimized these primers for determining their optimal annealing temperature. For RT-PCR, the primer sets used were as follows: IL-11 forward, 60-AGCTGCAAGGTCAAGATGGT; IL-11 reverse, 60-TCCTTAGCCTCCCTGAATGA; IL-15 forward, 60-TGGATGCAAAGAATGTGAGG; IL-15 reverse, 60-TTGAAATGCCGAGTGTTTTG; GAPDH forward, 60-CCTGCACCACCAACTGCTTA; and GAPDH reverse, 60-CATGAGTCCTTCCACGATACCA. Usage of the ABI PRISM 7000 Sequence Detection System made it possible to measure the amount of mRNA in the sample.

***Statistical analysis***

Experimentation was done three times, and Student’s t-test (**P<0.01, ***P<0.001) calculated the significance using the Graph-Pad Prism 5.01 software. Checking for statistical significance between the two groups was made successful by merely using the t-test. The data normality was checked beforehand. For this purpose, the test of Shapiro Wilk was used for each pair under consideration. For *in vivo* study Friedman and Kendall’s W test was run separately between two groups at a time. Multiple comparisons were performed to check the significance between patient groups.

## Results

***Reduction in IL-15 and IL-11 expression post in vitro HBV infection***

**Figure 1:**
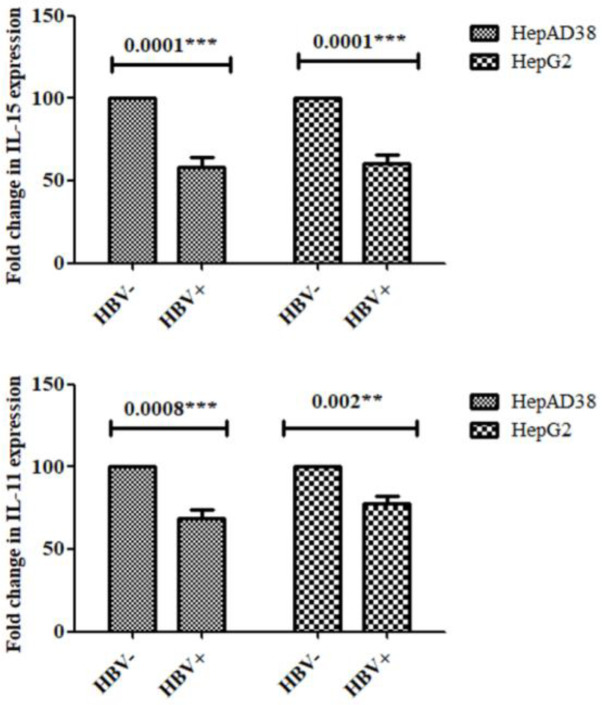
*In vitro* IL-15 and IL-11 down-regulation mRNA expression post HBV infection

The* in vitro *studies showed that IL-15 and IL-11 expression mRNA was considerably decreased in HBV replicating and HBV-induced cells in contrast with control cells. This was seen in Figure 1.

***In vitro*** **Effect of Anti-oxidant (NAC) and calcium modulators treatment on IL-15 production**

As the infection of HBV *in vitro* (HepAD38 and HepG2) as well as *in vivo *resulted in reduced expression of IL-15 and IL-11, we further checked if the treatment of immune modulators can elevate the expression of both cytokines. 

**Figure 2:**
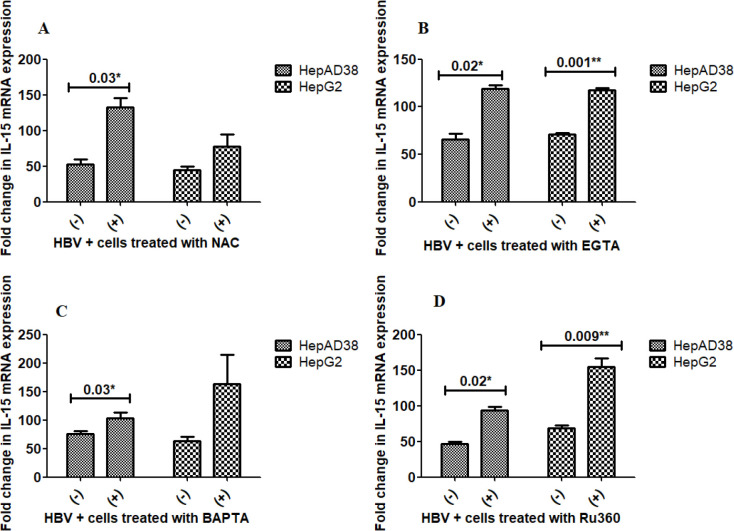
Effect of NAC, EGTA, BAPTA, and Ru360 treatment on mRNA expression of IL-15

Results indicate that a trend in up-regulation of IL-15 mRNA expression was observed in both cell lines after the treatment with all stated modulators (Figure 2A-D). The difference in the IL-15 levels was significant between EGTA as well as Ru360 treatments in both cell lines (Figures 2B and 2D), but it was not significant between NAC and BAPTA treatments in either cell line (Figures 2A and 2C).

***In vitro*** **effect of Anti-oxidant (NAC) and calcium modulators on IL-11 production**

**Figure 3:**
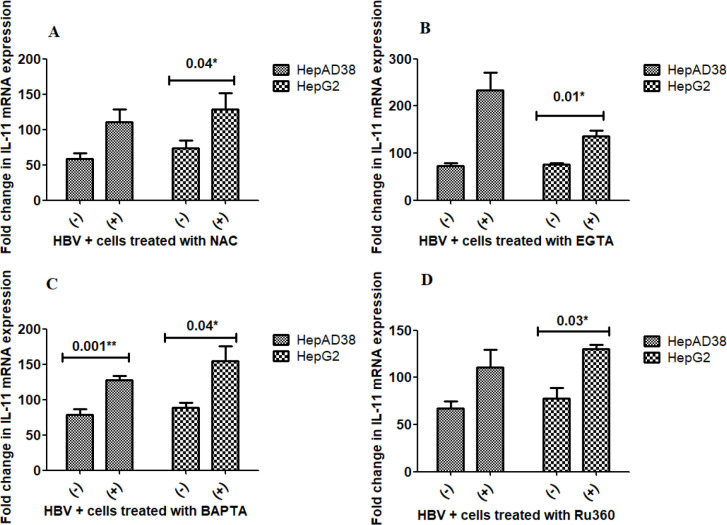
Effect of NAC, EGTA, BAPTA, and Ru360 treatment on mRNA expression of IL-11

The *in vitro* effect of HBV infections as well as NAC and calcium blockers on the expression of IL-11 was also evaluated. Similar to the increase in IL-15 mRNA expression seen in both cell lines after treatment with the NAC, calcium chelators (EGTA-AM, BAPTA-AM) and calcium blocker Ru360, a trend was observed in the up-regulation of IL-11 mRNA expression in both HBV replicating and induced cells HepAD38 and HepG2, respectively. Significant up-regulation was observed in the HepG2 cell line after treatment with NAC (*P*=0.04), EGTA-AM (*P*=0.01), BAPTA-AM (*P*=0.04), and Ru360 (*P*=0.03) treatment (Figure 3A-D). In the HepAd38 cell line, a significant difference after treatment was only observed with BAPTA (*P*=0.001) (Figure 3C).

***Differential expression of IL-15 and IL-11 among control, inactive carrier, recovered cases, and chronic HBV patients***

**Figure 4:**
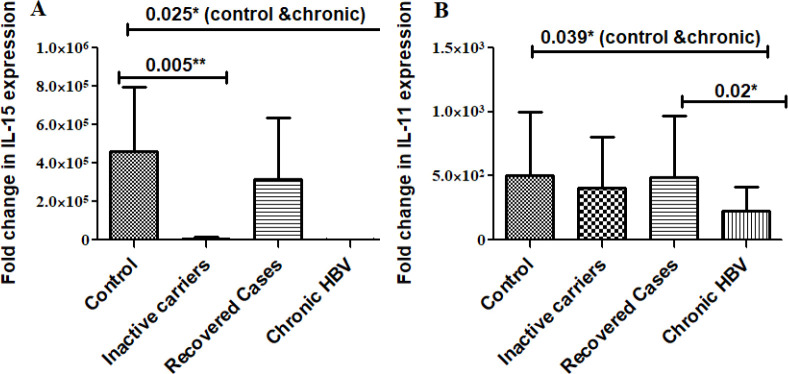
Differential expression of IL-15 and IL-11 among various HBV-infected patient groups

Real-time PCR quantified the *in vivo* expression of IL-15 and IL-11. Control, inactive HBV carriers, HBV recovered, and chronic hepatitis B patients, (Figures 4A and 4B) several comparative analysis shows that IL-15 was significantly down-regulated in HBV-infected groups (inactive carriers, *P*=0.005; and chronic HBV infected, *P*=0.025) compared with uninfected control. IL-11 was significantly down-regulated in the chronic HBV-infected groups (*P*=0.039) in contrast with the uninfected control as well as recovered cases (*P*=0.02).

Overall these results indicate that HBV infection tends to decrease the expression of these cytokines (as in cases of inactive carriers and chronic HBV infected group in contrast with healthy controls) while recovery from the infection tends to restore the expression of both cytokines, indicating a positive effect of these cytokines’ expression on overall diseases state. 

HepAD38 cells 3 days post HBV induction while HepG2 cells were processed 5 days post HBV induction. The results were compared. The accuracy, as well as reliability of data, was confirmed by using GADPH as an internal control. IL-15 and IL-11 mRNA expression were significantly reduced in infected cells in contrast with non-infected control cells. Student’s t-test determined the significance of the difference. The X-axis shows the HBV negative cells and HBV positive cells

(A-D) HBV + cells treated with 250 μM NAC, 5 µM EGTA, and 5 µM BAPTA for 24 hr while 10 µM Ru360 treatment was given for 12 hr, respectively. A strong up-regulating trend was observed in both cell lines after the treatment with all stated modulators. Although the difference was significant in the case of EGTA (*P*=0.02*, 0.001**) and Ru360 (*P*=0.02*, 0.009**) for both of the cell lines (2B and 2D) while NAC (*P*=0.03*) and BAPTA (*P*=0.03*) treatment resulted in a significant increase of IL-15 expression only in HepAD38 cell line (2A and 2C). The X-axis indicates the HBV+ cells with and without treatment, and on the Y-axis, both cell lines show the relative levels of IL-15. Using student’s t-test, the result’s significance was determined

** (**A-D**) **HBV + cells treated with 250 μM NAC, 5 µM EGTA, and 5 µM BAPTA for 24 hr while 10 µM Ru360 treatment was given for 12 hr. A trend of up-regulation of IL-11 mRNA expression was observed in both cell lines post-NAC, calcium chelators (EGTA-AM, BAPTA-AM), and calcium blocker Ru360 process. Although, significant up-regulation was observed in the HepG2 cell line after NAC (*P*=0.04*), EGTA-AM (*P*=0.01*), BAPTA-AM (*P*=0.04*), and Ru360 (*P*=0.03*) treatment (3A-D), while in HepAd38 cell line, significant difference post-treatment was observed for BAPTA only (*P*=0.001**) (3C). The X-axis indicates the HBV+ cells with and without treatment and the Y-axis shows the relative expression levels of IL-11 in both cell lines. Using student’s t-test, the result’s significance was determined

Friedman and Kendall’s W test was run separately between two groups at a time. (A) For IL-15 the overall significance is 0.062*. Multiple comparison analysis indicates that IL-15 was significantly down-regulated in HBV-infected groups (chronic HBV *P*=0.005**, inactive cases *P*=0.025*) in contrast with the uninfected control. (B) For IL-11 the overall significance is 0.095*. IL-11 was also down-regulated in the chronic HBV-infected groups (*P*=0.039*) in contrast with the uninfected controls and recovered cases (*P*=0.02*) significantly. The X-axis depicts varied group samples and the Y-axis is the relative value of the expression

## Discussion

Cytokines and chemokines have fundamental roles in activating, sustaining, and coordinating immunological homeostasis along with inflammatory mechanisms. Accounting for the cytokine as well as the chemokine profiles within patients contributes a valuable clue about the disease condition. Inflammatory responses are involved in causing infiltration and withholding of immune cells at some stage during liver damage. It is reported that the release of cytokines results in non-cytolytic intracellular viral deactivation and might play a significant part during the clearance of HBV without killing infected cells (26). Thus serum cytokine levels and changes in these cytokines during the course of infection are indicative of inflammatory changes going on in the liver and elsewhere, as a result of HBV infection. 

IL-15 and IL-11 are important cytokines of immunity, their role during inflammation is under critical scientific investigation. Investigating the IL-15 and IL-11 expression levels during the course of HBV infection is critically significant so that their modulation mechanism may be exploited for designing new therapeutic approaches for either viral control and/or viral clearance.

In the current study, IL-15 and IL-11 differential expression pattern in various HBV-infected groups and *in vitro *restoration of their expression by treatment with various calcium modulators and anti-oxidants were investigated. 

As compared with uninfected control cells,* in vitro* studies showed that within both cell lines, IL-15 and IL-11 mRNA expression was significantly down-regulated. Compared with uninfected control in accordance with *in vitro *outcomes, it was also explored that IL-15 and IL-11 *in vivo* expression was also down-regulated within HBV-infected patients (Figure 4). Therefore overall, HBV infection resulted in decreased expression of both cytokines *in vitro* as well as *in vivo. *

Various viruses induce the expression of IL-15 which then plays an important role in inducing the antiviral responses by various lymphocyte populations (27, 28). The effect of IL-15 on the outcome of viral infections greatly varies from virus to virus. For NK- as well as CD8 T-cell-controlled viral diseases, these may be exacerbated IL-15 deficiency, while loss of IL-15 may help viral infection that causes lymphoproliferative dysfunction (27, 28). Infected patients with hepatitis B have also been reported to induce expression of IL-15 and its low levels. Low disease progression and high viremia are connected to circulating IL-15 (29). Our results are in line with these observations. Lowering levels of IL-15 may indicate a general problem or a reduction in the quantity, functionality, or reactivity of IL-15-producing cells. Therefore, the risk of the virus taking control is associated with the production of IL-15 below ideal levels, most likely as a result of the lack of protective NK cell responses to persist in the absence of IL-15. IL-11 viral infections have previously been related to IL-11 expression (30, 31). The liver is one tissue that expresses IL-11 and its receptors (32). Chronic inflammatory disorders, lipopolysaccharide-induced sepsis, macrophage inflammation, nephrotoxic nephritis, and T-cell-mediated liver injury are all reduced by IL-11 treatment (33).

Thus, a decreased level of IL-11 observed in our study, both *in vivo* and *in vitro*; indicates high levels of inflammation after HBV infection both* in vivo *and *in vitro*. 

Restoration of the normal cytokine production using various chemical modulators was then investigated *in vitro*. NAC, various anti-oxidants, and calcium chelators have previously been reported to have antiviral effects in the case of HIV, HCV, and HBV by modulating various downstream signaling pathways. However, no study as yet has reported if the observed antiviral effect of these modulators has some impact on IL-15 and IL-11 production. Therefore IL-15 and IL-11 production effects, both pre and post-treatment with NAC and calcium modulators were evaluated. 

Our results indicated that comparing HBV-infected controls that were not treated with NAC *in vitro*, the mRNA expression of IL-15 was considerably up-regulated. *In vitro *NAC treatment significantly up-regulated the mRNA expression of IL-15 as compared with non-treated HBV-infected controls. Treatment with EGTA-AM and Ru360 raised IL-15 mRNA expression significantly in both cell lines while the increase was significant with NAC and BAPTA-AM treatment only in HepAD38 cells. 

In all cases, expression of both cytokines was restored/up-regulated post modulator treatment indicating the overall positive effect of treatments. The *in vivo* results indicate that inactive carriers and chronic HBV-infected individuals have relatively lower levels of these cytokines as compared with recovered cases as well as healthy controls. Thus overall, an increase in the expression using calcium and anti-oxidants should restore normal immune function and virus clearance. Various previous studies indicate that NAC (34-36) and calcium modulators (12, 37-43) have positive effects on the expression of various cytokines and disease outcomes. 

The study provides insight into the importance of the stated cytokines and their role in HBV pathogenesis and disease progression. However, the data needs to be validated in a larger study cohort with more patients and control samples in each group. Furthermore, the qRT-PCR results should be validated at the protein level using techniques such as ELISA or western blot. 

## Conclusion

These findings hold significant importance for the therapeutic interventions given to HBV-infected patients at various stages of disease progression. Further work is required to determine whether this increase or decrease in cytokine production is due to the direct or indirect effect of NAC, EGTA-AM, BAPTA-AM, and Ru360 treatment. Thus, the current study, in this regard, provides a new avenue of possibility in direct therapeutic interventions enhancing the cytokine production, for HBV infection clearance /management that often progresses to liver cancer. 

## Authors’ Contributions

KJ, AJ, and SS conceived and designed the experiments. KJ collected the patient’s samples and patient data and performed the experiments. KJ, AJ, SS, and AW analyzed the data. KJ, SS, and AW contributed reagents and analysis tools. KJ, AJ, SS, and AW wrote the paper. All authors read and approved the final manuscript.

## Funding

This research was funded by the Higher Education Commission of Pakistan (HEC) under the IRSIP program (IRSIP 35 BMS 22), NUST student funds of Dr Aneela Javed, and IIUI student funds of Dr Shaheen Shahzad.

## Coflicts of Interest

The authors have no conflicts of interest. 
